# Early-Life Exposure to the Chinese Famine and Risk of Cognitive Decline

**DOI:** 10.3390/jcm8040484

**Published:** 2019-04-10

**Authors:** Hongguo Rong, Xiaozhen Lai, Elham Mahmoudi, Hai Fang

**Affiliations:** 1 China Center for Health Development Studies, Peking University, Beijing 100083, China; hgrong@hsc.pku.edu.cn (H.R.); laixiaozhen@pku.edu.cn (X.L.); 2 Department of Family Medicine, University of Michigan, Ann Arbor, MI 48109, USA; mahmoudi@med.umich.edu

**Keywords:** China, cognitive decline, fetal, childhood, famine

## Abstract

Previous studies on the Chinese famine suggested long-term effects of early-life famine exposure on health conditions. This study aims to investigate the association between exposure to the Chinese famine of 1959–1961 at different early-life stages and the risk of cognitive decline in adulthood. A total of 6417 adults born between 1952 and 1964 in the 2015 survey data of China Health and Retirement Longitudinal Study were included in this study. Cognitive performance was estimated through a series of comprehensive neuropsychological tests, including the Telephone Interview of Cognitive Status (TICS-10), word recall, and pentagon drawing. Multiple generalized linear model (GLM) was employed to detect the association between multi-stage early-life famine exposure and late-life cognitive performance. Compared with the unexposed group, respondents exposed to famine in the fetal period performed worse in the TICS (difference −0.52, 95% confidence interval (CI): −0.93 to −0.10), word recall (difference −0.46, 95% CI: −0.74 to −0.19), and general cognition (difference −1.05, 95% CI: −1.64 to −0.47). Furthermore, we also found negative effects of famine exposure on performance of word recall and pentagon drawing in the early (word recall difference −0.56, 95% CI: −1.00 to −0.11; pentagon drawing difference −0.76, 95% CI: −1.40 to −0.12), mid (word recall difference −0.46, 95% CI: −0.81 to −0.11; pentagon drawing difference −0.66, 95% CI: −1.16 to −0.16), and late (word recall difference −0.30, 95% CI: −0.55 to −0.04; pentagon drawing difference −0.75, 95% CI: −1.13 to −0.37) childhood-exposed groups. Early-life famine exposure in different stages is positively associated with late-life cognitive decline. Fetal famine exposure might affect the overall cognitive status in adulthood, and childhood famine exposure has potential adverse effects on visuospatial episodic memory.

## 1. Introduction

Cognitive impairment and Alzheimer’ disease (AD) are alarming public health issues, causing harm to patients’ health, longevity, well-being, and quality of life. Additionally, they imply heavy economic burdens for patients and their care-givers across the world, including China [1]. The developmental origins of health and disease (DOHaD) hypothesis proposed that adverse prenatal and postnatal living conditions as well as subsequent mismatched environment may increase the risk of neurodegenerative disorders and chronic diseases [2]. Indirect evidence in support of this hypothesis stems from studies which proposed that AD-related brain pathological changes might originate at the very beginning of life [3,4,5], and that fetal or childhood nutrition play an irreplaceable role in healthy brain development [6].

As a “natural experiment”, famine provides an acquirable setting to study the association between nutritional deprivation in early life and chronic or neurodegenerative diseases, especially age-related cognitive disorders. Despite strong evidence indicating the importance of early-life nutrition on long-term health and well-being [7], studies have shown conflicting results on the effects of early-life famine exposure on late-life cognitive performance. Some Dutch famine studies reported that fetal famine exposure was neither associated with mental retardation and intelligence quotient score in early adulthood [8], nor with the cognitive performance in adulthood [9]. However, de Rooij adopted a cognitive battery test to estimate cognitive function at ages 56 to 59, and suggested that early-gestation famine exposure was negatively associated with selective attention ability [10]. Therefore, the association between fetal nutrition and adult cognitive decline is still in question. Furthermore, recent studies have shifted their attention onto the long-term adverse effects of malnutrition in childhood, but no consistent conclusions have yet been drawn. One possible explanation may lie in the difference in childhood stages when exposed to the famine [11]. Visuospatial episodic memory, as a critical component of cognitive function, involves encoding, storage and retrieval of external or internal information [12]. Studies suggested that episodic memory developed dramatically in childhood and adolescence, during which hippocampal changes might play a critical role [13,14]. However, little is known about the long-term effects of childhood malnutrition on episodic memory or other subdivisions of cognition.

The Chinese famine of 1959–1961 is recognized as one of the severest catastrophic events in Chinese history. Compared with other famines, such as the 1944–1945 Dutch famine and the 1932–1933 Holodomor famine, it was of prolonged duration (3 years), with a wider scope, and threatened countless lives in the country [15]. The Chinese famine provides a unique opportunity to observe the long-term negative effect of fetal and childhood malnutrition, as both stages are at crucial periods of physical development and mental health.

Early life undernutrition has resulted in serious health consequences across the world, especially in many developing countries. It was estimated recently that undernutrition in fetal period and childhood cause 3.1 million child deaths annually, counting for 45% of overall child mortality [7]. Therefore, the prevention of early-life malnutrition is still a major public health concern, and early-life interventions are recommended to help malnourished children.

Today, most Chinese people in their mid-fifties or older were once exposed to the 1959–1961 famine in their prenatal or postnatal period. Faced with the rapid aging trend in China, and the lasting impact of early-life undernutrition in many developing countries across the world, it is urgent to examine the link between prenatal and postnatal famine exposure and the risk of adult cognitive decline.

## 2. Methods

### 2.1. Study Population

This study used data from 2015 China Health and Retirement Longitudinal Study (CHARLS) to investigate the association between fetal/childhood famine exposure and adult cognitive decline. CHARLS is an ongoing, nationwide representative longitudinal cohort study of Chinese adults aged 45 years and older [16]. The CHARLS surveys, applying a face-to-face computer-assisted personal interview (CAPI), took place from June 2011 to March 2012, with follow-up surveys every two years [17]. The initial baseline survey of CHARLS included 17,708 respondents in 150 counties/districts and 450 villages or residential communities from 28 provinces [18]. The 2015 wave is the latest follow-up survey, and sampled 21,095 respondents. The CHARLS surveys serve as an excellent nationwide sample to represent the middle-aged and elderly people in China. In this study, we analyzed individual-level data from the latest 2015 follow-up survey to investigate the famine effects on late life cognitive decline. The program protocol was in line with the Declaration of Helsinki and was ethically approved by the Biomedical Ethics Review Committee of Peking University (approval number: IRB00001052–11015) [16].

### 2.2. Famine Cohorts and Area Categories

**Figure 1:**
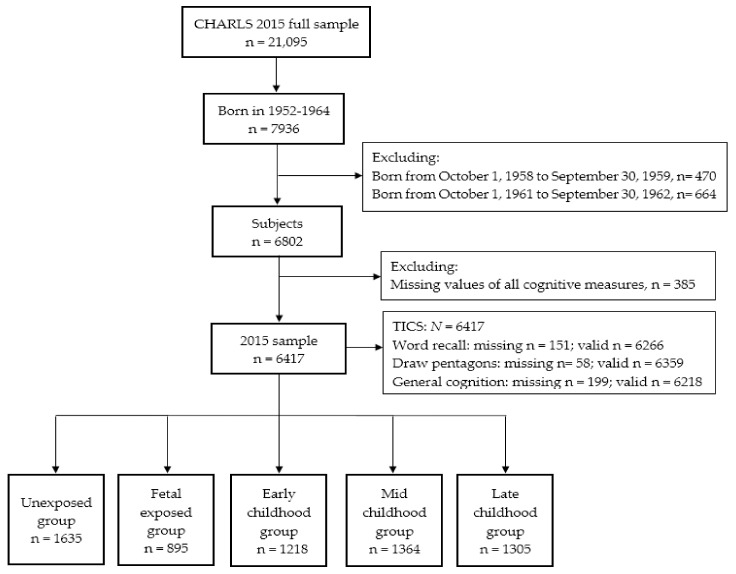
Flow chart of the sample selection from 2015 China Health and Retirement Longitudinal Study (CHARLS) survey. TICS: Telephone Interview of Cognitive Status.

Figure 1 shows the flow chart of sample selection in this study. It has been widely accepted that the Chinese famine, which began in 1959 and lasted for three years until 1961, affected all regions of mainland China [19,20]. Due to the unclear start and end dates of this famine, respondents born from 1 October 1958 to 30 September 1959, and from 1 October 1961 to 30 September 1962 were excluded to minimize misclassification. After excluding respondents lacking values of all cognitive measures, a total of 6417 respondents were included in this study. To make this study as comparable with existing studies [21,22] as possible, we adopted the most commonly used definition of famine exposure and categorized respondents into five groups based on their date of birth: the unexposed group (born from 1 October 1962 to 30 September 1964, *n* = 1635; born at least one year after the end of famine), the fetal-exposed group (born from 1 October 1959 to 30 September 1961, *n* = 895; respondents born during the famine), the early childhood-exposed group (born from 1 October 1956 to 30 September 1958, *n* = 1218; respondents aged 1–2 years old when the famine started), the mid childhood-exposed group (born from 1 October 1954 to 30 September 1956, *n* = 1364; respondents aged 3–4 years old when the famine started), and the late childhood-exposed group (born from 1 October 1952 to 30 September 1954, *n* = 1305; respondents aged 5–6 years old when the famine started).

The famine brought poverty, hunger and pains to the entire mainland China, but the severity of damage varied regionally. This study used the excess death rate (EDR) of each province to determine the severity of the famine, which was calculated as the percentage change in mortality rate from the mean level in 1956–1958 to the highest value in 1959–1961 [23]. Taking 100% EDR as the threshold, severely or less severely famine-affected areas were determined at the provincial level, and respondents in each of the five exposure cohorts were divided into severely or less severely affected populations. The CHARLS survey asked respondents the provinces where they lived during their childhood, so we were able to identify whether they were born in the severely famine-affected provinces or not. Meanwhile, the CHARLS structured questionnaire recorded respondents’ general health status in childhood and Hukou status (urban/rural; the Hukou system refers to the national household registration that restricted free migration from rural to urban areas [24]), which allowed us to control for famine, regions, and birth cohorts.

### 2.3. Assessment of Cognitive Performance

A comprehensive neuropsychological test was adopted to assess the respondents’ cognitive performance. Consistent with Health and Retirement Study (HRS) in the United States and previous CHARLS studies on cognition [25,26,27], this study measured cognitive performance by three tests, including Telephone Interview for Cognitive Status (TICS-10, attention and orientation), word recall (episodic memory), and pentagon drawing (visuospatial ability).

Telephone Interview for Cognitive Status captures individual’s mental status by asking ten questions, including five items from the serial subtraction of 7 from 100 (up to five times) and five items on time orientation (recalling the year, month and day of today’s date, day of the week, and season of the year). The TICS score equals the number of correct answers, ranging from zero to ten (Cronbach’s alpha = 0.8) [17].

Word recall aims to assess the respondent’s episodic memory in cognition. The score in this section, ranging from zero to ten, was measured by the average score of immediate and delayed recalls of ten Chinese words [28].

As to pentagon drawing, respondents were asked to redraw a picture of two overlapped pentagons. Those who successfully redrew the picture scored one, while those who failed scored zero. This test was used to estimate respondent’s visuospatial skills [29].

The general cognition, measured by the sum of all three sectional scores with a range of zero to twenty-one, is considered as a comprehensive reflection of one’s overall cognitive status.

### 2.4. Assessment of Covariates

The CHARLS structured questionnaire conducted face-to-face interviews to record respondents’ information on socioeconomic status (SES), demographics, health habits and clinical characteristics. Covariates included education level (primary school and below, junior school, high school, college and above), marital status (married and living with spouses, or otherwise), smoking (smokers or non-smokers), drinking (drinker or non-drinker), Hukou status (rural or urban), depression, activities of daily living (ADLs), self-reported general health status (excellent, very good, good, fair, and poor), health status in childhood (excellent, very good, good, fair, and poor), and self-reported doctor-diagnosed chronic disease (hypertension, diabetes, dyslipidemia, and heart diseases). Heart diseases include heart attack, coronary heart disease, congestive heart failure, angina, and other heart problems. The 10-item Center for Epidemiological Studies Depression Scale (CES-D-10) was used to estimate respondents’ depression symptoms, ranging from zero to 30 [30]. ADLs in CHARLS are evaluated with a 6-term summary assessed with an ADL scale that includes bathing, dressing, eating, getting in/out of bed, using the toilet, and controlling urination [31]. The respondents classified as ADL-independent had the ability to complete all of the six activities without difficulty, whereas those who had difficulty in any items were regarded as ADL-impaired.

### 2.5. Statistical Analysis

Continuous variables with normal distribution were described as means ± SDs, and those with non-normal distribution were presented as medians (interquartile ranges, IQRs). Discrete variables were shown as percentages (%). We used analysis of variance or Kruskal–Wallis test to compare continuous variables, and chose Pearson′s chi square test to estimate the difference among proportions. The Bonferroni corrections were adopted to perform post-hoc comparisons with unexposed group.

In the cross-sectional design of the current study, the association of fetal and multi-stage childhood famine exposure (0, 1, 2, 3, 4 were assigned to unexposed, fetal period, early, mid, and late childhood-exposed groups, respectively) with late-life cognitive performance was estimated by multiple generalized linear model (GLM) to control for potential confounders, including age, gender, famine severity, education level, marital status, drinking, ADLs, depression, self-reported general health status, health status in childhood and self-reported doctor-diagnosed chronic diseases. Coefficients with 95% confidence intervals (CIs) were reported. All statistical analyses were conducted using STATA, version 13.0 (Stata Corp, College Station, TX, USA).

## 3. Results

### 3.1. Characteristic Description

Table 1 shows the basic characteristics of the respondents in this study. In all, 3117 males and 3300 females were included in the analytical sample. Among the overall cohort (*n* = 6417), 895 (13.9%) respondents had fetal exposure to Chinese famine, and 3887 (60.9%) respondents were exposed to the famine during their childhood. The early life-exposed groups had a lower education level than that in the unexposed group. Compared with the unexposed group, the childhood-exposed groups had lower proportion of married respondents living with spouses, and higher prevalence of ADL impairment and heart disease. Moreover, respondents in the mid childhood-exposed group had higher prevalence of ADLs impaired and hypertension, and those in late childhood-exposed group tended to have a lower frequency of self-reported good health status, and higher scores on depression symptom, as well as a higher ADLs impaired prevalence. Although the prevalence of diabetes and dyslipidemia in famine-exposed groups were higher than that in the unexposed group, no significance was shown after Hochberg modification of Bonferroni corrections.

### 3.2. Early-Life Famine Exposure and Late-Life Cognitive Performance

Table 2 provides the results of cognitive measures (TICS, word recall, pentagon drawing, and general cognition) in each group. Compared with the unexposed group, respondents in childhood-exposed groups had lower scores of TICS, word recall, pentagon drawing, and general cognition, while in the fetal-exposed group, significance was not noticed in the pentagon drawing test.

Table 3 shows the relationship between Chinese famine exposure and subsequent cognitive decline in multiple GLM. Compared with the unexposed group, respondents exposed to famine in fetal period performed worse in TICS scores (difference −0.52, 95% confidence interval (CI): −0.93 to −0.10; *p* = 0.015), word recall (difference −0.46, 95% CI: −0.74 to −0.19; *p* = 0.001), and general cognition (difference −1.05, 95% CI: −1.64 to −0.47; *p* < 0.001) after adjusting for age, gender, famine severity, education level, marital status, drinking, activities of daily living (ADLs), depression, self-reported general health status, health status in childhood and self-reported doctor-diagnosed chronic disease. Furthermore, we also found negative effects of famine exposure on performance of word recall and pentagon drawing in the early (word recall difference −0.56, 95% CI: −1.00 to −0.11; *p* = 0.014; pentagon drawing difference −0.76, 95% CI: −1.40 to −0.12; *p* = 0.020), mid (word recall difference −0.46, 95% CI: −0.81 to −0.11; *p* = 0.010; pentagon drawing difference −0.66, 95% CI: −1.16 to −0.16; *p* = 0.010), and late (word recall difference −0.30, 95% CI: −0.55 to −0.04; *p* = 0.022; pentagon drawing difference −0.75, 95% CI: −1.13 to −0.37; *p* < 0.001) childhood-exposed groups.

### 3.3. Association between Famine Severity and Cognitive Performance

Table 4 shows the results of stratified analysis by famine severity. In order to isolate the potential confounding effect of aging on the famine-cognition association, the study classified the respondents into the severely-affected famine group and less severely-affected famine group. In severely famine-affected areas, the childhood-exposed respondents had lower scores in word recall, pentagon drawing, and general cognition tests than those in the unexposed group. However, no significance was observed in less severely-affected areas. Furthermore, the fetal-exposed group had worse performance in word recall (difference −0.48 95% CI −0.94 to −0.02, *p* = 0.039). In less severely-affected areas, only the fetal-exposed group had worse performance in TICS, word recall, and general cognition. As Table 4 indicated, the adjusted model of GLM showed poorer performance of early-exposed groups in word recall, pentagon drawing, and general cognition in severely famine-affected areas as compared to the unexposed group.

## 4. Discussion

Using a nationally representative sample of 6417 community-dwelling Chinese adults, the association between early-life famine exposure and cognitive function was examined. Three main findings emerged: (1) extreme fetal and childhood malnutrition has a strong association with adult cognitive function; (2) fetal famine exposure might affect overall cognitive status and childhood famine exposure could worsen individual’s visuospatial episodic memory; and (3) the early-exposed groups in severely famine-affected areas had poorer performance in word recall, pentagon drawing and general cognition than those in less severely famine-affected areas.

This study provides new evidence to the hypothesis that intrauterine and childhood malnutrition has a long-term influence on cognitive performance [32,33]. Fetal period and infancy are crucial periods for normal brain formation and development. Nutritional deficiencies during these early stages may influence brain function, cognition, and behavior throughout the lifespan [32]. The evidence from Dutch famine studies suggested that malnutrition at the very beginning of life did longstanding harm to mental and physical health. Dutch famine birth cohort study suggested that only in males, prenatal malnutrition was associated with lower physical performance (β = −0.8, 95% CI: −1.5 to 0.0) and grip strength (β = −4.2, 95% CI: −8.2 to −0.3) [34]. Studies also showed that early gestation malnutrition had an adverse effect on the selective attention performance of both males and females at the age of 56 to 59, but concerning general cognition, memory function, and perceptual motor learning, similar associations did not appear [10]. Compared with the 1944–1945 Dutch famine, Chinese famine had higher mortality (an estimated 30 million premature deaths) and longer duration (persisting from 1959 to 1961) [19]. Unlike studies on Dutch famine which only focused on malnutrition in fetal period [35,36], the Chinese famine provides a unique opportunity to explore the long-run effect of multi-stage early-life famine exposure on late-life cognitive function. Xu et al. used the 2011–2013 CHARLS sample of 2446 rural respondents to study the famine–cognition association in six year cohorts (born between 1958 and 1963) [26], suggesting a poorer TICS performance in the 1961 cohort (those who experienced full-term prenatal and one-year postnatal famine exposure) and a negative famine effect on the longitudinal change in general cognition during the 2-year follow-up in the 1959 cohort compared with the 1963 cohort (unexposed group). This is the first study to investigate the famine-cognitive association in a non-European population, but only rural respondents are included, which limits the generalizability of their main findings because the Chinese famine affected not only rural but also urban regions in mainland China [19]. Xu et al. included those born in the same year in to a group, while this study classified respondents in a different way based on the most common definition of famine exposures. In this study, two time points (1 October and 30 September in each year) were set to determine each group, and those born from 1 October 1958 to 30 September 1959, and from 1 October 1961 to 30 September 1962 were excluded to minimize misclassification. Some respondents born in the early 1959 were actually conceived in 1958 and thus did not experience prenatal famine exposure, while post-famine cohort respondents conceived in 1961 and born in the early 1962 experienced partial-term prenatal famine exposure. In this study, we improved the generalizability of our findings by setting multi-stage childhood famine-exposed cohorts.

The current study suggested that respondents exposed to famine during fetal period or early childhood had a poorer cognitive performance than those in unexposed group. After adjusting for potential confounding risk factors in the multiple GLM (Table 3), we found an association between multi-stage childhood famine exposure and an increased risk of cognitive decline. There is no doubt that intrauterine nutritional status also plays a crucial role in brain development, but the results in this study indicated that not only the fetal period, but also early childhood stage might be the critical “window” period for brain development. Researchers have demonstrated that human brain began to form at about two weeks’ postconceptional age, and to reach 80% of its adult size by the first two years after birth [33]. Animal studies have proved the importance of adequate nutrition during gestation and infancy for the neurodevelopmental process, such as neuronal proliferation, differentiation and migration [37,38]. Meanwhile, a 47-year longitudinal Barbados Nutrition Study demonstrated that childhood malnutrition was associated with personality disorder symptoms and physical and mental health in adulthood [39]. In line with the Ghana famine study [40], our results also suggest that childhood is a critical stage during which malnutrition may have an irreversible adverse effect on cognition. Therefore, the nutritional status in childhood, especially in the first two years after birth, is particularly important for physical and mental growth and cerebral development.

Cognition is a complex system which involves multiple domains, including episodic memory, executive function, working memory, attention, and information-processing speed [41]. Our findings reveal that different stages of early-life exposure to the Chinese famine might affect different cognition domains in adulthood: while fetal exposure may influence the overall cognitive status, childhood exposure may do harm to visuospatial episodic memory. In line with the findings of Xu et al. [26], we found that fetal famine exposure was associated with poorer performance in TICS, word recall and general cognition, while childhood famine exposure was linked to worse performance in word recall and pentagon drawing. The TICS test (screening time orientation and attention) in CHARLS chiefly detects respondents’ executive function to reflect their mental status. The word recall test primarily assesses the respondent’s episodic memory, and the pentagon drawing test is used to estimate visuospatial skills. The combination of the TICS test (date naming and counting backwards from 100, subtracting 7 each time) and the pentagon drawing test can be considered as a subset of mini-mental state examination (MMSE) [42], which is commonly used to estimate respondent’s overall cognitive status. Our findings confirmed the results of a previous study which adopted MMSE to assess the overall cognitive status, digit symbol subtest of WAIS-RC to measure executive function, and auditory verbal learning test (AVLT) to estimate the episodic memory [43]. Some other studies on the Chinese famine also held similar opinions [44,45]. Interestingly, we found that childhood famine exposure was associated with worse performance in visuospatial episodic memory (Table 3), and the early childhood-exposed group in severely famine-affected areas had poorer performance in visuospatial episodic memory and overall cognitive status than those in less severely famine-affected areas (Table 4). Visuospatial episodic memory develops rapidly during early childhood [46], but the reason for this development has not yet been fully understood. Research suggests that structural connectivity between the hippocampus and lateral parietal regions is relevant to the episodic memory development in childhood [46,47,48,49]. Our findings demonstrate the long-term negative influence of childhood nutritional deprivation on adult visuospatial episodic memory. The famine-cognition association is a multidimensional phenomenon, influence by variables such as timing, duration, famine severity, and special measures of cognition [50]. Meanwhile, the cognitive neuroscience is still in its early infancy, so further population-based investigations on early-life detection of cognitive decline or clinical magnetic resonance imaging (MRI) are necessary to further elucidate this association. Aging is a known risk factor for the progression of cognitive decline. In order to isolate the effect of aging from the association between famine exposure and late life cognitive function, we conducted a stratified analysis by famine severity, and found worse cognitive performance of early-exposed groups in severely famine-affected areas compared to that of the unexposed group in the adjusted model of GLM (Table 4). To some extent, it can be interpreted that the severer malnutrition experienced in early life, the more serious cognitive decline occurred in later life.

In order to isolate the potential confounding effect of aging on the famine-cognition association, the study classified the respondents into the severely-affected famine group and less severely-affected famine group. In severely famine-affected areas, the childhood-exposed respondents had worse performance in word recall, pentagon drawing, and general cognition tests than those in the unexposed group. However, no significance was observed in less severely-affected areas. Furthermore, the fetal-exposed group had worse performance in word recall (difference −0.48 95% CI −0.94 to −0.02, *p* = 0.039). In less severely-affected areas, only the fetal-exposed group had worse performance in TICS, word recall, and general cognition. As Table 4 indicated, the adjusted model of GLM showed poorer performance of early-exposed groups in word recall, pentagon drawing, and general cognition in severely famine-affected areas than the unexposed group.

What attributes to the association between early-life famine exposure and cognitive decline later in life? In the past decades, epidemiological studies have demonstrated that severe malnutrition during prenatal and postnatal stages may increase the risk of chronic non-communicable diseases (NCDs) [51,52]. Recent studies on the Chinese famine shows an increased risk of dyslipidemia, hypertension, and metabolic syndrome [20,53,54]. Our findings also support the hypothesis that fetal and childhood malnutrition could elevate the risk of hypertension, diabetes and dyslipidemia at adulthood. These NCDs are recognized as the dominant factors of cognitive decline among the elderly, which could affect brain structure and might lead to age-related cognitive disorders. Studies on the Dutch famine suggest that early gestation malnutrition contributes to more atherogenic lipid profiles in adulthood [55,56]. Another Chinese famine study also found the association between early famine exposure and higher risk of dyslipidemia [20]. Experimental evidence proved that 27-hydroxycholesterol, a cholesterol-oxidized metabolite, may play a role in the development of mild cognitive impairment (MCI) because it can facilitate β-amyloid peptide (Aβ) formation and accumulation in brain [57,58].

The present study has a few limitations. First, as an age-related disorder, cognitive decline worsens with age. However, the prolonged Chinese famine affected almost the whole China [59], making it impossible to identify a control group not affected by famine in 1959–61. Second, there is potential recall bias of self-reported early-life health status, potentially resulting in an underestimation of the famine–cognition relationship [60]. Third, birth weight is an important indicator of early-life nutrition. However, birth records are not well preserved, so the data on birth weight and height could not be collected for this study. Fourth, we did not have information on dietary intake, which is a major indicator of “rich” nutritional status in adulthood, and thus a potential confounder for cognitive decline. Finally, the government reports from which we obtained provincial EDR may be subject to falsification. Even so, EDR and cohort size shrink age indices (CSSI) are still the most commonly used measurements of famine severity in Chinese famine studies [19]. To partially minimize the influence of these limitations, this study classified the respondents into severely and less severely-affected groups, and adjusted for age in the multiple GLM regression model, so as to better test the hypothesis of joint effects of early-life malnutrition and later life environment on adult cognitive function. It is necessary to explore the relationship between early-life malnutrition/dietary factors and risk of cognitive decline later in life. Today, there are still a large number of children in the world that are at risk of malnutrition, which may put them at risk of future cognitive decline. The results from the present study can be used in other countries in which malnutrition still is an issue among children, and help them improve children’s cognition. Potential confounders in both childhood and late life should be carefully adjusted to minimize the residual confounding. More chronic diseases (such as cancers) should be measured and reported carefully and accurately in the survey data. Other cognitive functions, such as language production and feelings, may be also needed to measure the comprehensive status of cognitive functions for famine-exposed populations.

## 5. Conclusions

In conclusion, this national cohort study suggests that famine exposure during fetal period and childhood is associated with overall cognitive status and visuospatial episodic memory in adulthood, respectively. Our study stresses on the importance of nutrition in both fetal period and childhood on adult cognitive functions. Thus, the promotion of early-life nutritional interventions to help achieve healthy aging and mitigate cognitive decline in later life is worth consideration.

## Figures and Tables

**Table 1:** General characteristics of the respondents in the five Chinese famine-exposed groups.

	Unexposed	Fetal Exposed	Childhood-Exposed		
			Early Childhood	Mid Childhood	Late Childhood
*N*	1635	895	1218	1364	1305
Birth date ^a^	1962–1964	1959–1961	1956–1958	1954–1956	1952–1954
Age in 2015	52.0 (0.7)	55.1 (0.7) *	58.0 (0.7) *	60.0 (0.7) *	62.0 (0.7) *
Male, *n* (%)	796 (48.7)	414 (46.3)	612 (50.3)	668 (49.0)	627 (48.1)
Severely-affected area, *n* (%)	—	290 (32.4)	444 (36.5)	544 (39.9)	522 (40.0)
Education, *n* (%)					
Primary school and below	730 (44.7)	427 (47.7)	711 (58.4)	885 (64.9)	971 (74.4)
Junior school	603 (36.9)	246 (27.5)	292 (24.0)	299 (21.9)	219 (16.8)
High school	206 (12.6)	181 (20.2)	170 (14.0)	140 (10.3)	77 (5.9)
College and above	94 (5.8)	41 (4.6)	44 (3.6)	39 (2.9)	38 (2.9)
Smoking, *n* (%)	462 (28.3)	261 (29.2)	374 (30.7)	429 (31.5)	389 (29.8)
Drinking, *n* (%)	641 (39.2)	345 (38.6)	473 (38.9)	490 (36.0)	444 (34.0) *
Marital status	1557 (95.2)	837 (93.5)	1133 (93.0) *	1246 (91.4) *	1164 (89.2) *
Hukou status, *n* (%)					
Rural	1207 (77.7)	645 (76.4)	898 (77.2)	1015 (78.7)	965 (78.6)
Urban	347 (22.3)	199 (23.6)	265 (22.8)	274 (21.3)	263 (21.4)
Self-reported good health status, *n* (%)	243 (14.9)	115 (12.9)	134 (11.0)	167 (12.2)	128 (9.8) *
Good health status in childhood (self-reported), *n* (%)	315 (19.5)	182 (20.6)	246 (20.4)	248 (18.4)	243 (18.9)
Depression, median (IQR)	6 (3, 11)	6 (3, 11)	6 (3, 11)	6 (3, 11)	6 (3, 12) *
ADL-impaired, *n* (%)	184 (11.3)	128 (14.3)	206 (16.9) *	215 (15.8) *	283 (21.7) *
Hypertension, *n* (%)	391 (26.2)	248 (30.9)	343 (30.6)	400 (31.7) *	456 (37.6) *
Diabetes, *n* (%)	123 (8.4)	90 (11.2)	122 (11.0)	128 (10.2)	136 (11.4)
Dyslipidemia, *n* (%)	268 (18.4)	177 (22.5)	223 (20.3)	250 (20.1)	256 (21.8)
Heart disease, *n* (%)	196 (13.2)	128 (15.9)	200 (18.0) *	223 (17.7) *	246 (20.4) *

^a^ From 1 October year to 30 September year. ADLs: activities of daily living. Continuous variables are expressed as median (interquartile range), and categorical variables are shown as *n* (%). The Kruskal–Wallis test was used for continuous variables with a skewed distribution, and the Pearson’s chi squared test for categorical variables when comparing differences. * *p* < 0.0125 (after Bonferroni correction).

**Table 2:** Performance of cognitive measures in five Chinese famine-exposed groups.

	Unexposed	Fetal Exposed	Childhood-Exposed		
			Early Childhood	Mid Childhood	Late Childhood
TICS (range: 0–10)	7.1 ± 2.6	6.7 ± 2.7 *	6.5 ± 2.8 *	6.6 ± 2.8 *	6.2 ± 2.9 *
Word recall (range: 0–10)	4.0 ± 1.7	3.7 ± 1.7 *	3.4 ± 1.8 *	3.4 ± 1.7 *	3.3 ± 1.7 *
Draw pentagons (binary: 0/1)	1257 (77.4%)	652 (73.1%)	809 (67.1%) *	873 (64.8%) *	778 (60.3%) *
General cognition (range: 0–21)	11.9 ± 3.7	11.2 ± 3.9 *	10.7 ± 4.0 *	10.7 ± 4.0 *	10.2 ± 4.2 *

TICS: Telephone Interview of Cognitive Status. Data are shown as mean ± standard and *n* (%). Analysis of variance or the Kruskal–Wallis test was used for continuous variables with a normal distribution and a skewed distribution, and the Pearson χ2 test for categorical variables when comparing differences. * *p* < 0.0125 (after Bonferroni correction).

**Table 3:** Multiple GLM estimates the association of famine exposure with cognitive decline in 2015.

	Unexposed	Fetal Exposed	Childhood-Exposed		
			Early Childhood	Mid Childhood	Late Childhood
TICS (range: 0–10)					
Coef.	Ref.	−0.52 (−0.93, −0.10)	0.22 (−0.46, 0.90)	0.33 (−0.20, 0.87)	0.01 (−0.40, 0.38)
*p*		0.015 *	0.518	0.225	0.972
Word recall (range: 0–10)					
Coef.	Ref.	−0.46 (−0.74, −0.19)	−0.56 (−1.00, −0.11)	−0.46 (−0.81, −0.11)	−0.30 (−0.55, −0.04)
*p*		0.001 *	0.014 *	0.010 *	0.022 *
Draw pentagons (binary: 0/1)					
Coef.	Ref.	−0.40 (−0.83, 0.03)	−0.76 (−1.40, −0.12)	−0.66 (−1.16, −0.16)	−0.75 (−1.13, −0.37)
*p*		0.066	0.020 *	0.010 *	< 0.001 *
General cognition (range: 0–21)					
Coef.	Ref.	−1.05 (−1.64, −0.47)	−0.75 (−1.71, 0.21)	−0.41 (−1.17, 0.34)	−0.54 (−1.08, 0.01)
*p*		< 0.001 *	0.124	0.282	0.053

Data are shown as coefficient with 95% confidence intervals. TICS: Telephone Interview of Cognitive Status; Coef: coefficient; Ref.: reference group. Model adjusted for age, gender, education, marital status, famine severity, drinking, activities of daily living, depression, self-reported general health status, health status in childhood, hypertension, and heart disease. * *p* < 0.05.

**Table 4:** Association between famine exposure and cognitive decline stratified by famine severity.

	Unexposed	Fetal-Exposed	Childhood-Exposed		
			Early Childhood	Mid Childhood	Late Childhood
TICS					
Severely	Ref.	−0.13 (−0.81, 0.55)	−0.47 (−1.50, 0.57)	−0.20 (−1.01, 0.61)	0.01 (−0.59, 0.60)
*p*		0.707	0.375	0.629	0.980
Less	Ref.	−0.77 (−1.29, −0.24)	0.79 (−0.12, 1.68)	0.73 (−0.02, 1.44)	−0.00 (−0.51, 0.51)
*p*		0.005 *	0.090	0.043 *	0.996
Word recall					
Severely	Ref.	−0.48 (−0.94, −0.02)	−0.95 (−1.65, −0.26)	−0.89 (−1.44, −0.35)	−0.76 (−1.16, −0.36)
*p*		0.039 *	0.007 *	0.001 *	<0.001 *
Less	Ref.	−0.43 (−0.77, −0.09)	−0.27 (−0.85, 0.31)	−0.16 (−0.62, 0.30)	0.02 (−0.31, 0.35)
*p*		0.013 *	0.355	0.491	0.905
Draw pentagons					
Severely	Ref.	−0.07 (−0.19, 0.05)	−0.19 (−0.37, −0.01)	−0.23 (−0.37, −0.08)	−0.22 (−0.32, −0.11)
*p*		0.239	0.041 *	0.002 *	<0.001 *
Less	Ref.	−0.05 (−0.15, 0.04)	−0.09 (−0.25, 0.07)	−0.04 (−0.16, 0.09)	−0.07 (−0.16, 0.02)
*p*		0.254	0.253	0.558	0.124
General cognition					
Severely	Ref.	−0.69 (−1.67, 0.28)	−1.75 (-3.22, −0.28)	−1.48 (-2.64, −0.32)	−1.09 (−1.94, −0.25)
*p*		0.162	0.020 *	0.012 *	0.011 *
Less	Ref.	−1.26 (−2.00, −0.52)	−0.00 (−1.26, 1.26)	0.36 (−0.64, 1.34)	−0.14 (−0.85, 0.57)
*p*		0.001 *	1.00	0.487	0.703

Data were shown as coefficient with 95% confidence intervals. TICS: Telephone Interview of Cognitive Status; Ref.: reference group. Model adjusted for age, gender, education, marital status, famine severity, drinking, activities of daily living, depression, self-reported general health status, health status in childhood, hypertension, and heart disease. * *p* < 0.05.
